# Nitrous Oxide Emission and Denitrifier Abundance in Two Agricultural Soils Amended with Crop Residues and Urea in the North China Plain

**DOI:** 10.1371/journal.pone.0154773

**Published:** 2016-05-06

**Authors:** Jianmin Gao, Yingxin Xie, Haiyang Jin, Yuan Liu, Xueying Bai, Dongyun Ma, Yunji Zhu, Chenyang Wang, Tiancai Guo

**Affiliations:** 1 National Key Laboratory of Wheat and Maize Crop Science/Collaborative Innovation Center of Henan Grain Crops/National Engineering Research Center for Wheat, Henan Agricultural University, Zhengzhou, 450002, China; 2 College of Resources and Environment, Henan Agricultural University, Zhengzhou, 450002, China; Institute of Genetics and Developmental Biology, CHINA

## Abstract

The application of crop residues combined with Nitrogen (N) fertilizer has been broadly adopted in China. Crop residue amendments can provide readily available C and N, as well as other nutrients to agricultural soils, but also intensify the N fixation, further affecting N_2_O emissions. N_2_O pulses are obviously driven by rainfall, irrigation and fertilization. Fertilization before rainfall or followed by flooding irrigation is a general management practice for a wheat-maize rotation in the North China Plain. Yet, little is known on the impacts of crop residues combined with N fertilizer application on N_2_O emission under high soil moisture content. A laboratory incubation experiment was conducted to investigate the effects of two crop residue amendments (maize and wheat), individually or in combination with N fertilizer, on N_2_O emissions and denitrifier abundance in two main agricultural soils (one is an alluvial soil, pH 8.55, belongs to Ochri-Aquic Cambosols, OAC, the other is a lime concretion black soil, pH 6.61, belongs to Hapli-Aquic Vertosols, HAV) under 80% WFPS (the water filled pore space) in the North China Plain. Each type soil contains seven treatments: a control with no N fertilizer application (CK, N0), 200 kg N ha^-1^ (N200), 250 kg N ha^-1^ (N250), maize residue plus N200 (MN200), maize residue plus N250 (MN250), wheat residue plus N200 (WN200) and wheat residue plus N250 (WN250). Results showed that, in the HAV soil, MN250 and WN250 increased the cumulative N_2_O emissions by 60% and 30% compared with N250 treatment, respectively, but MN200 and WN200 decreased the cumulative N_2_O emissions by 20% and 50% compared with N200. In the OAC soil, compared with N200 or N250, WN200 and WN250 increased the cumulative N_2_O emission by 40%-50%, but MN200 and MN250 decreased the cumulative N_2_O emission by 10%-20%. Compared with CK, addition of crop residue or N fertilizer resulted in significant increases in N_2_O emissions in both soils. The cumulative N_2_O emissions from the treatments of 250 kg N ha^-1^ were 1.1–3.3 times higher than those of treatments with 200 kg N ha^-1^ in both soils with adding equal amounts of the same type of crop residue. Abundance of the 16S rRNA gene did not significantly change in all treatments in two soils, but the *nosZ* and *nirS* genes were more abundant in soils amended with crop residues compared with CK or N-only treatments. N_2_O emission, however, were not related to the abundance of denitrifier containing nirS or nosZ. The research provided some information regarding the effect of crop residues with N fertilizer on N_2_O emissions and denitrifier abundances in two soils. Our results imply the property of crop residue and rate of N fertilizer are important influencing factors of N_2_O emission when crop residues combined with N fertilizer are applied to different agricultural soils.

## Introduction

Agricultural soils are a significant source of greenhouse gases, mainly because they are responsible for more than 50% of anthropogenic nitrous oxide (N_2_O) emissions [1]. N_2_O production in soils originates primarily from microbially-mediated nitrification and denitrification processes which are affected by soil type, soil water content, Oxygen (O_2_) availability, nitrogen (N) fertilizer application, organic carbon (C) content, and other parameters [2].

Crop residue amendments can provide readily available C and N, as well as other nutrients to agricultural soils [2], but also intensify the N fixation and biological N binding [3], which affects N_2_O emissions [4–7]. In China, the total amount of crop residue production has been estimated to be 600–800 million tons per year, with wheat and maize straw accounting for 25~40% [8], with considerable amounts of crop residues remaining in the fields after harvest. Incorporating crop residues into the field has been highly recommended as a measure to promote organic matter recycling and for environmentally friendly, sustainable agricultural production [9, 10]. The Chinese government has banned the burning of crop straws, and this has resulted in the increasing use of wheat, maize, and rice straw as soil amendments in China [11]. Some below relative tillage practices are made usually to mix the residue and fertilizer in this region as the following steps: straw returning after crop harvest in last season, and then fertilization, and taking rotary tillage, especially for wheat season. In maize season, residue and N fertilizer were not sometimes in the same soil layer, but mixed application of fertilizer and straw were also frequently encountered in the some deep plowing and subsoiling farmland.

China is one of the major agricultural countries in the world. To increase grain yields, inputs of chemical fertilizer (500–700 kg N ha^-1^year^-1^) [12, 13] have far exceeded the optimum or recommended fertilizer levels (127–350 kg N ha^-1^year^-1^) [14–16]. Of all the types of chemical N fertilizer used, urea is the most common, accounting for 60%-66% of the total fertilizer N used in China [17].

The combined application of chemical fertilizer and crop residues is considered to be beneficial for improving soil fertility and nutrient utilization, but also influences N_2_O emissions and the abundance of denitrifying microbes in soil [4]. Crop residues and soil type are important factors affecting N_2_O emission [18]. In China, the application of crop residues combined with N fertilizer has been broadly adopted in agricultural areas, but the effects of this cultivation practice on N_2_O emissions from agricultural soil remains largely unstudied in the North China Plain which is the major wheat and maize production region in China. In the region, a lime concretion black soil (pH 6.61, belongs to Hapli-Aquic Vertosols, HAV) and an alluvial soil (pH 8.55, belongs to Ochri-Aquic Cambosols, OAC) are the two most common soil types, comprising 384.88 million ha and 3.224 million ha, respectively.

Literature reviews have suggested that the abundance of soil denitrifier communities may be factors that affect denitrification. The nirK, nirS and nosZ genes were usually used as genetic marker to investigate denitrifying community abundance. The common difference between true denitrifiers and other microorganisms with nitrate-reducing ability is that the true denitrifiers have either a copper-containing nitrite reductase encoded by nirK or a cytochrome cd1 nitrite reductase encoded by nirS. The nitrite reductase can reduce nitrite (NO_2_) to nitric oxide (NO) which is considered to be the key step of the denitrification pathway because the reduction of NO_2_ to NO is the first step to produce a gaseous product. N_2_O reductase encoded by nosZ gene can catalyse the reduction of nitrous oxide to molecular nitrogen, which is the last step in the complete denitrification pathway. The nosZ gene is missing in approximately one-third of the denitrifying bacteria. The denitrifier community abundances from some agricultural soils have been quantified based on nirK, nirS and nosZ genes [4, 19]. Nevertheless, attempts to identify the influences of organic fertilizer on the denitrifier community abundance in different soil types and agroecosystems have little shown consensus in recent studies [20]. Recent reports have shown that the properties and uses of soil are important factors that affect the diversities of nitrifiers and denitrifiers [20, 21]. However, to date, few studies have evaluated the influence of crop residue amendments on N_2_O emissions and denitrifier community abundance in the HAV and OAC soils.

N_2_O is mainly emitted from the agricultural soils as pulses after strong rainfall, irrigation and N fertilization. Contribution of pulse values to total emission is more than 70% for the agricultural soils in the North China Plain[22, 23]. Fertilization before rainfall or followed by flooding irrigation is a general management practice in this area, but this practice would provide abundant N substrate and create temporal anaerobic condition for denitrification. The water filled pore space (WFPS) is less than 50% under the conditions without rainfall and irrigation, but with rainfall or irrigation, the WFPS values of more than 70% are frequent at this area[23]. In agricultural soils, N_2_O is mostly thought to originate from nitrification when the soil moisture content is below 60% WFPS, while denitrification dominates as source at soil moisture content exceeding 60% WFPS. Smith and Tiedje [24] suggested that it was important to understand the denitrification response to increasing soil moisture, in order to better characterize denitrification N losses from field soils following rainfall or irrigation. Therefore, understanding the dynamics of N_2_O emission and denitrifier abandance is essential to find a way of N_2_O emission mitigation after rainfall and irrigation in the North China Plain. But little information is currently available concerning the impact of crop residues with inorganic fertilizer on N_2_O emission and denitrifier abundance from the soil of winter wheat-maize rotation under high soil moisture content.

The objective of this study was to investigate the effects of different crop residue amendments, individually or combined with or without N fertilizer, on (1) N_2_O emissions from two main agricultural soils under high soil moisture content in the North China Plain; and (2) total bacterial and denitrifier abundance in the two soil types.

We hypothesized that (1) crop residue amendments combined with high rate of N fertilizer would increase N_2_O production, conversely, decrease N_2_O production in comparison to N fertilizer addition alone and the control, and (2) crop residue ammentments should lead to increase of the denitrifier abundance in comparison to N fertilizer addition alone and the control.

## Materials and Methods

### Soil sampling

Soil samples were collected from the 0–20 cm layer of the two soil types (an alluvial soil, pH 8.55, belongs to Ochri-Aquic Cambosols, OAC, the other is a lime concretion black soil) after summer maize harvest from the fields in which winter wheat and summer maize were cultivated in a one-year rotation system. The OAC soil was collected from the experimental farm at Henan Agricultural College (113.59E, 34.86N) in Zhengzhou, Henan, China on september 26th, 2014, and the HAV soil was collected within Zhumadian city (114.53E, 33.41N), Henan, China on september 28th, 2014. The OAC soil derived from Northwest Loess Plateau, which was rich in calcium carbonate of loess sediments with groundwater depth and about 1 g/L mineralization degree. The annual mean temperature in this region is 14.1°C, and the annual mean rainfall is 641 mm. The HAV soil derived from quaternary lacustrine sediments on the semi hydromorphic soil, more than 40% montmorillonite clay mineral in 0-40cm tillage layer of this soil with less coarse sand usually leads to the too sticky and the large expansion coefficient in soil texture. The annual mean temperature in this region is 14.8°C, and the annual mean rainfall is 852 mm. Texture and properties of the both soils were in Table 1.

**Table 1:** Physical and chemical characteristics of the soils used in this study.

Soil types	Sand (g kg^-1^)	Slit (g kg^-1^)	Clay (g kg^-1^)	Organic C (g kg^-1^)	Total N (g kg^-1^)	pH (1:1 soil and water)
Ochri-Aquic Cambosols	755	100	132	9.99	0.82	8.55
Hapli-Aquic Vertosols	393	350	245	16.2	0.96	6.61

Soil samples were kept frozen at -20°C prior to measuring soil NO_3_^-^ concentration. One day before conducting the experiments, the soil samples were thawed. The soils were then homogenized, passed though a 2-mm sieve, and stored in the dark at 4°C. Maize and wheat residues were both sampled at second day after harvest at the experimental farm at Henan Agricultural College (113.59E, 34.86N) in Zhengzhou, Henan, China. Both crop residues included all above-ground material. Crop residues were dried at 55°C continually for 24 hours and ground to pass through a 2-mm sieve before being mixed into the soil. The total C and N of crop residues was determined by dry combustion using a CNS elemental analyzer (LECO). The amounts of cellulose, hemicellulose, and lignin of crop residues were measured with the acid detergent fiber method[25]. Some chemical properties of crop residues used in the experiment are shown in Table 2.

**Table 2:** Some chemical properties of crop residues used in the experiment.

Crop residue	Total C %	Total N	C/N	Cellulose %	lignin	Hemicellulose
Maise residue	44.9	0.86	52	31.5	24.1	27.4
Wheat residue	39.7	0.41	97	20.6	52.1	23.7

### Experimental design

The experiments included two N fertilizer levels and two crop residues. There were seven treatments for each soil type, consisting of a control with no N fertilizer application (CK, N0), 200 kg N ha^-1^ (N200), 250 kg N ha^-1^ (N250), maize residue plus N200 (MN200), maize residue plus N250 (MN250), wheat residue plus N200 (WN200) and wheat residue plus N250 (WN250). All treatments were replicated three times. All treatments was added to achieve a WFPS of 80% by urea in distilled water solution, which was equal to the average WFPS of the region after rainfall or irrigation. The required dry soil to fill the columns to 15 cm depth, along with the corresponding crop residues, were weighed into the separate jars with 20 cm diameter. The tops of the jars were covered using PVC caps that had a 10-mm hole for aeration and reduction of soil moisture loss. Five grams maize or wheat residue per 1 kg dry soil corresponding to 4404 and 3731 kg C ha^-1^, respectively, was added and fully mixed. The crop residue application rates were based on currently practices and the amounts of crop residues after harvest. The incubation jars were stored at 24°C ± 1°C in thermostatic chambers throughout the 30-day incubation period. Soil water content was maintained at 80% WFPS by regular addition of distilled water.

Gas samples were collected at 0, 1, 2, 3, 6, 9, 12, 15, 18, 21, 24, 27, and 29 days to analyze N_2_O after the trial commencement. At each sampling time, the top cap was sealed with a glass stopper which have a triple valve for 60 min. Then gas from the headspace was sampled using a 25-ml gas-tight plastic syringe at 0, 30 and 60min after sealing. Prior to sampling the headspace, the internal air was mixed by pumping the syringe twice to remove any stratification. The gas sample was injected into a pre-evacuated glass vial. The jars were opened after gas sampling, and 25 g of soil was sampled to analyze soil NO_3_^-^–N and NH_4_^+^–N. Cumulative N_2_O emissions were calculated by linearly interpolating the natural log (Ln)-transformed gas emission values between the two measurement periods. At days 0, 1, 3, 12, 18, 24, and 29, soil samples were collected to determine nitrifier and denitrifier community abundances. The N_2_O concentration was simultaneously analyzed with an Agilent 7890A autosystem gas chromatograph fitted with an electron capture detector and a merchandiser and flame ionization detector (Agilent Technologies, California, USA). The NO_3_^-^–N and NH_4_^+^–N in the soil were extracted using a 1:5 ratio of soil:2 M KCl solution, and the filtered extracts were analyzed calorimetrically (UV6300, Mapada Instruments, Shanghai, China).

### DNA extraction and real-time quantitative PCR

The soil samples in 15-ml tubes were freeze-dried overnight until completely dried prior to nucleic acid extraction. The freeze-dried soil was stored at -80°C. Nucleic acids were extracted from 0.5 g samples of freeze-dried soil with the FastDNA^®^ SPIN Kit (America Mobio) following the manufacturer’s instructions. Extracted DNA was visualized by agarose (1.5% w/v) gel electrophoresis. The recovered DNA was eluted in 10 mM Tris-HCl buffer (pH 7.5). The purity and the quantity of the DNA samples were determined with a UV Spectrophotometer at 260 and 280 nm. The A_260/280_ ratios were all >1.8. The DNA-containing solutions were stored at -20°C.

Total bacterial abundances were determined using the real-time quantitative TaqMan PCR method as described by Suzuki et al. [26]. The primers BACT1369F (5’-CGGTGAATACGTTCYCGG-3’), PROK1492R (5’-GGWTACCTTGTTACGACTT-3’) and probe TM1389 (5’-CTT GTA CAC ACC GCC CGTC-3’)[26] were used. The 20-μl amplification mixtures contained 10 μl of TaqMan Universal PCR Master Mix, 0.4 μl of each primer at 20 mM, 8 μl of H_2_O, 0.2 μl of the probe and 1 μl of template DNA. Thermal cycling conditions for amplification of the 16S rRNA genes were as follows: pre-incubation at 94°C for 5 min, 40 cycles consisting of denaturation at 94°C for 30 s, annealing at 55°C for 40 s, extension at 72°C for 30 s.

Abundances of nirS and nosZ genes were both determined using the SYBR green real-time qPCR method. The primer sets nirScd3aF:5’-AAC GYS AAG GAR ACS GG -3’ and nirS3cdR:5’-GAS TTC GGR TGS GTC TTS AYG AA -3’were used to apply the nirS gene fragment as described by Fabrizzi et al. [4]. The 20 μl reaction mixtures contained 10 μl of SYBR Green Premix ExTaq, 0.5 ul of each 20 mM primer, 7.8 ul of H_2_O, 0.2 μl 25 mM BSA and 1 μl of template DNA. The PCR conditions were as follows: 5 min at 95°C, and then 40 cycles consisting of 15 s at 95°C, 30 s at 55°C, and 30 s at 72°C. The primers used to amplify *nosZ* were nosZ1F: 5’-ATG TCG ATC ARC TGV KCR TTY TC-3’ and nosZ1R: 5’-WCS YTG TTC MTC GAC AGC CAG-3’[27]. The 20 μl-reaction mixtures contained 10 μl 2 x ABI Power SYBR I Green PCR Master Mix, 0.5 μl of each *nosZ*-specific primer, 8.3 ul of H_2_O, 0.2 μl 25 mM BSA and 0.5 μl of template DNA. Thermal cycling conditions for the nosZ primers were as follows: an initial cycle of 95°C for 10 min, then six cycles of 95°C for 15 s, 65°C for 30 s, 72°C for 30 s, followed by 40 cycles of 95°C for 15 s, 60°C for 15 s, 72°C for 30 s, and 83°C for 30 s.

The standard curves for the quantitative PCR assays were established using cloned 16S rRNA, *nirS* and *nosZ* gene fragments. The amplified 16S rRNA, *nirS* and *nosZ* gene fragments were gel-purified using the MiniBEST Agarose Gel DNA Extraction Kit Ver.3.0 (Takara Bio Inc), then cloned into the pMD^™^18-T vector using the pMD^™^18-T cloning kit (Takara Bio Inc) according to the manufacturer’s instructions. Plasmids were transformed into *Escherichia coli* JM109 competent cells. Plasmid DNA was extracted using the MiniBEST DNA Fragment Purification Kit Ver.3.0 and plasmid concentration was determined spectrophotometrically. Plasmid DNA was diluted in a ten-fold series to generate standard curves. All quantitative PCR reactions including unknown samples and standard curves were performed in triplicate.

### Statistical analysis

The software packages SPSS12.0 (SPSS Inc., Chicago, USA) and Excel 2007 (Microsoft Corporation, USA) were used for statistical data analysis. A general linear model for repeated measurements was applied to analyze the significance of the differences in the NH_4_^+^–N and NO_3_^-^–N contents, N_2_O emissions, and the abundance of denitrifiers between treatments. The differences in N_2_O fluxes between treatments were tested using one-way ANOVA. Nonlinear regression was used to describe the relationships between NH_4_^+^ and NO_3_^-^ contents and N_2_O emission and the denitrifier abundances. The significance of nonlinear regressions was determined using an *F*-test. Significances was accepted at a level of probability of *p*<0.05.

## Results

### N_2_O emission

**Fig 1:**
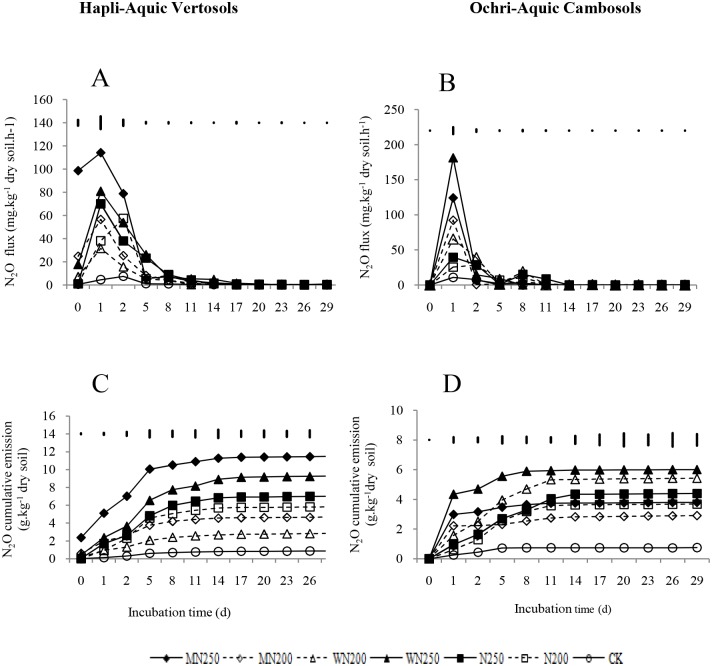
N_2_O flux (A, B) and cumulative N_2_O-N emission (C, D) from the Hapli-Aquic Vertosols and the Ochri-Aquic Cambosols soils. The vertical bars represent LSD_0.05_.

The N_2_O fluxes from all treatments except CK for the two soil types were obviously higher during the first two days of the incubation, but decreased rapidly thereafter (Fig 1). After six days of incubation, the N_2_O fluxes from all treatments almost reached the same level. The N_2_O flux from the MN250 treatment for the HAV soil and the WN250 treatment for the OAC soil reached their highest levels, 114.19 mg kg^-1^ h^-1^ and 181.15 mg kg^-1^ h^-1^, respectively, on the second day of incubation. At the start of incubation, the crop residue-amended treatments for both soils showed significantly enhanced N_2_O fluxes compared to those without crop residues.

The cumulative N_2_O emissions of the MN250 treatment with 11.49 g kg^-1^ was the highest for the HAV soil, while, for the OAC soil, the WN250 treatment with 6.01 g kg^-1^ was the highest (Fig 1). The cumulative N_2_O emissions from the N ferilizer-amended treatments with or without crop residues increased 3.2–13.1- and 3.9–8.0-fold compared to the CK for the HAV and OAC soils, respectively. It is noteworthy that cumulative N_2_O emissions from the treatments with 250 kg N ha^-1^ were 1.1–3.3 times higher than those from the treatments of 200 kg N ha^-1^ when equal amounts of the same type of crop residue were added to the two kinds of soil. In the HAV soil, MN250 and WN250 increased the cumulative N_2_O emissions by 60% and 30% compared with N250 treatment, respectively, but MN200 and WN200 decreased the cumulative N_2_O emissions by 20% and 50% compared with N200. In the OAC soil, compared with N200 or N250, WN200 and WN250 increased the cumulative N_2_O emission by 40%-50%, but MN200 and MN250 decreased the cumulative N_2_O emission by 10%-20%.

### Quantification of the 16S rRNA, nosZ, and nirS genes

**Fig 2:**
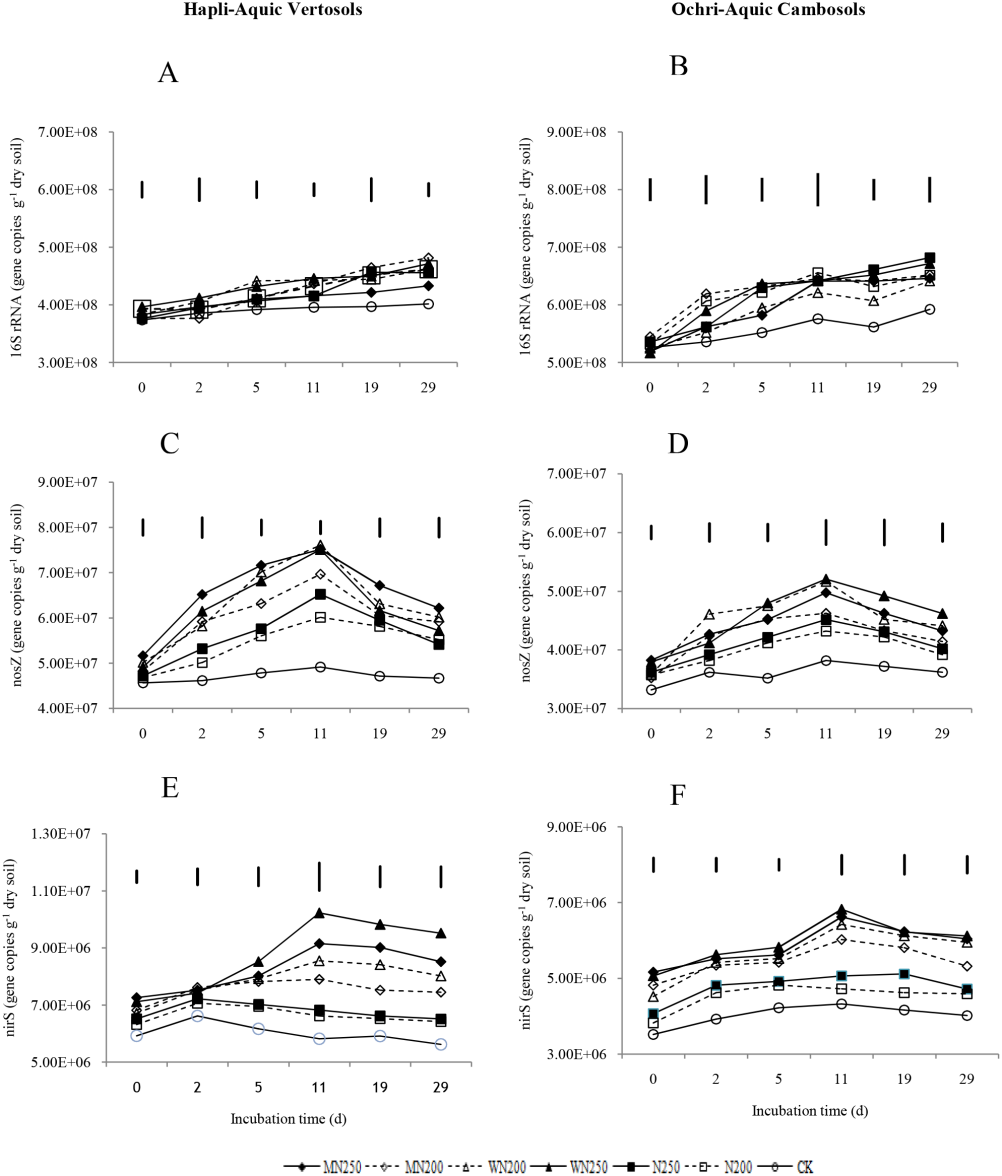
Abundances of the 16S rRNA (A, B), *nosZ* (C, D) and *nirS* (E, F) gene copies in the Hapli-Aquic Vertosols and the Ochri-Aquic Cambosols soils. The vertical bars represent LSD_0.05_.

The 16S rRNA gene abundances for all treatments did not obviously increase with incubation time (Fig 2). The changes in the *nosZ* and *nirS* gene abundances for all treatments were similar over the timecourse of incubation in both soils, increasing during the first period and then declining after 12 days (Fig 2). Addition of N fertilizer, with or without crop residues, resulted in an increase in the 16S rRNA, *nosZ* and *nirS* abundances compared to the CK for the two soils, but the abundances of the targeted genes did not significantly change among all the treatments. The *nosZ* abundances increased in all treatments from the first day to the 12th day and peaked at the 12th day, then slowly decreased in both soils. The average *nosZ* abundances in the N fertilizer- + crop residue treatments were higher than in the treatments without crop residues. The *nirS* gene copy numbers in the treatments containing crop residues were significantly higher than in the other treatments over the entire incubation time for both soils. The average *nirS* gene abundance in the MN250 treatment (7.91×10^6^ gene copies g^-1^ dry soil) was 1.6 and 1.3 times higher than in the CK (5.07×10^6^ gene copies g^-1^ dry soil) and the N250 treatment (5.7×10^6^ gene copies g^-1^ dry soil) in the HAV soil. In the OAC soil, the average *nirS* abundance in the W250 treatment (5.94×10^6^ gene copies g^-1^ dry soil) was 1.5 and 1.2 times greater than in the CK (3.95×10^6^ gene copies g^-1^ dry soil) and the N250 treatments (4.78×10^6^ gene copies g^-1^ dry soil), respectively.

### Soil mineral N

**Fig 3:**
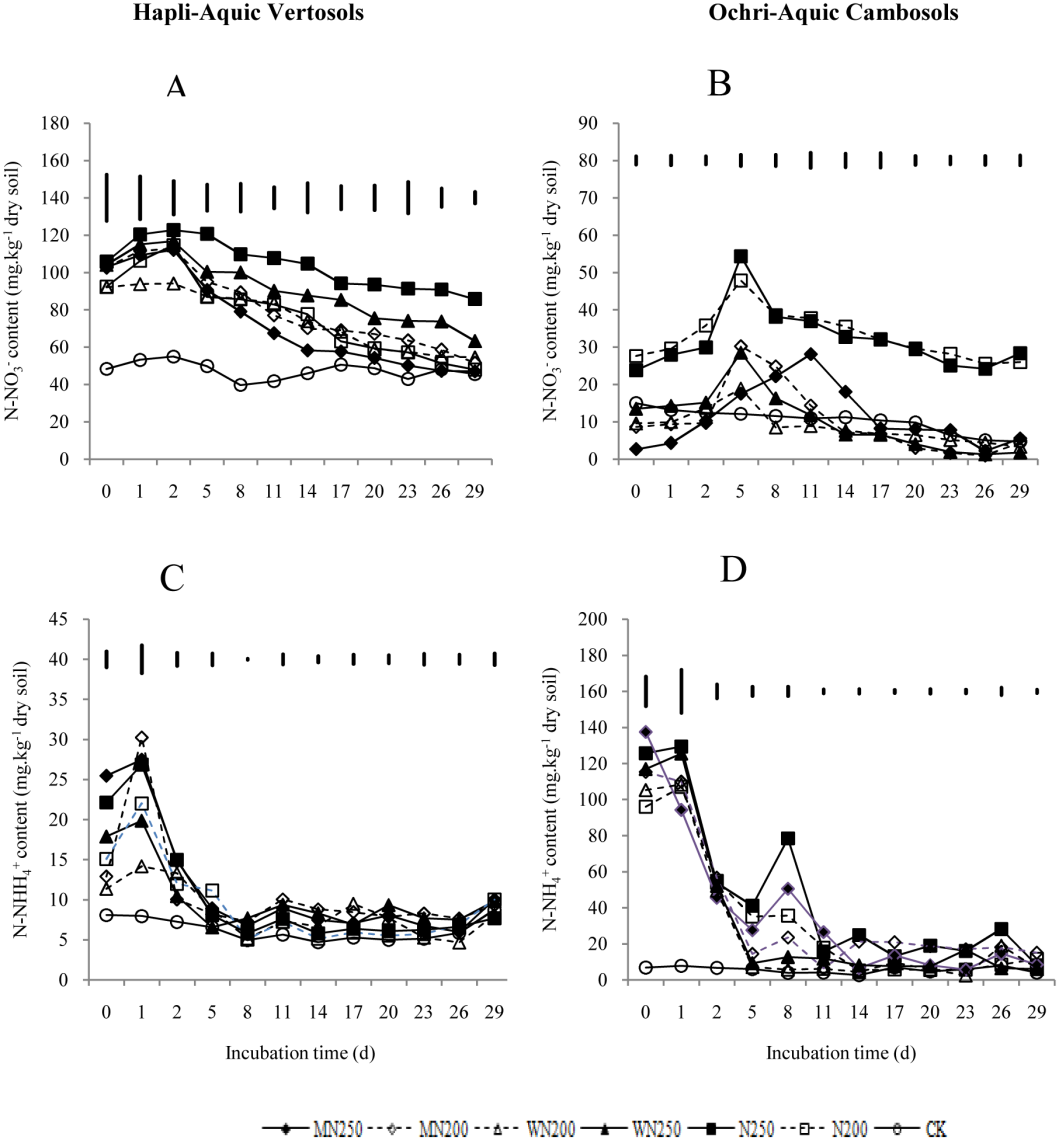
Dynamics of nitrate nitrogen (A, B) and ammonium nitrogen (C, D) contents in the Hapli-Aquic Vertosols and the Ochri-Aquic Cambosols soils. The vertical bars represent LSD_0.05_.

In the initial stages of the incubation, the N fertilizer-containing treatments, with or without crop residues, showed a significant increase in the content of ammonia nitrogen (NH_4_^+^–N) for both soils compared to the CK (Fig 3). The NH_4_^+^–N content of all treatments reached the maximum on the second day in the HAV soil, then decreased. There were no obvious differences between any of the treatments after three days of incubation. The MN200 treatment NH_4_^+^–N content maximumly changed during the incubation, from 30.24 g kg^-1^ to 9.30 g kg^-1^, while the W200 treatment showed a minimal change, from 14.17 g kg^-1^ to 8.10 g kg^-1^. In the OAC soil, there were no obvious differences in NH_4_^+^–N content for all the treatments. The average NH_4_^+^–N content for all treatments in the OAC soil was higher than for those in the HAV soil.

The nitrate nitrogen (NO_3_^-^–N) content of all treatments increased significantly in the first 12 days of incubation compared to the CK for the HAV soil, the N250 treatment was highest (Fig 3). In the OAC soil, the NO_3_^-^–N content of all treatments, except the MN250 treatment, increased significantly in the first six days, reaching a maximum on the sixth day, then decreasing. The NO_3_^-^–N content of the N fertilizer-only treatments was obviously higher than in the other treatments over the entire incubation period. In both soils, the average NO_3_^-^–N content of soil amended with only N fertilizer was higher than in those amended with a combination of crop residues and N fertilizer, suggesting incorporation of crop residues resulted in N immobilization. The average NO_3_^-^–N contents were higher for all the HAV soil treatments than they were for the OAC soil treatments.

## Discussion

### N_2_O emission

It has been well established that incorporation of crop residues can affect soil moisture, temperature, dissolved organic carbon concentration, inorganic N content, microbial activity, and redox potential [2], thus leading to regulation of N_2_O release in soil [28]. Inorganic fertilizer can provide abundant N to soil microorganisms, and further affect N_2_O emissions [21]. Previous studies have shown that N_2_O emissions from soils increase after the addition of plant residues [29–31] or application of inorganic fertilizer-N [31, 32]. In our study, N_2_O flux significantly increased compared to the CK after N fertilizer application either alone, or when combined with crop residues, for all treatments in the first two days of incubation, and reached the maximum on the second day, then rapidly decreased. Similar results were also obtained by other researchers [30, 33, 34]. This short-lived increase in N_2_O flux suggests that decomposition of N fertilizer and crop residues can provide a temporary abundance of C and N to microorganisms, and further, can directly stimulate microbial activity, resulting in a rapid increase in N_2_O emission [35]. With C and N consumption, the activity of microorganisms and subtrate N decreased, reducing the N_2_O to background levels. Variance analysis also shows crop residues combined N fertilizer significantly affect N_2_O cumulative emission (*P*<0.001).

A critical C:N ratio ∼30 has been accepted for predicting whether net N mineralization or net N immobilization occurs following crop residue addition, despite that this empirical parameter may vary slightly from one soil to another [36]. Plant materials of different C:N ratios could affect bacterial and fungal growth differently, thereby leading to considerable differences in the C:N ratio of newly produced microbial biomass [37, 38]. Crop residues with low C:N ratios (<30) in combination with inorganic N fertilizer can increase N_2_O emission, but inorganic N plus crop residues with C:N ratios >30 reduced N_2_O emissions [29]. Irrespective of soil aerations, soil N_2_O production was generally lower following plant materials with high C:N ratios than those with low C:N ratios [36]. Because it is generally recognized that incorporation of crop residues with high C/N ratio resulted in N immobilization, thus reducing concentrations of mineral soil N as substrate for N_2_O emission through nitrification and denitrification. However, in our study, an interesting result was obtained for maize residues, which had a lower C:N (52:1) than wheat residues (97:1), when amended with maize residues combined with N fertilizer, the cumulative N_2_O emissions were significantly reduced relative to the N fertilizer-only application in OAC soil, while wheat residue combined with N fertilizer led to a significant increase in cumulative N_2_O emission. The lower N_2_O emissions from maize residue treatments were likely caused by strong N immobilization. Wheat residue with higher ligin content degraded slower than maize residue, the slower decomposition likely meant little N immobilization [39], which can provide more available N in soil for denitrifier, further increase N_2_O emission. Similar unexpected results were also observed in other studies. Kong et al. [40] found that combined application of wheat residue (6 t ha^-1^, 174 kg N ha^-1^) and fertilizer-N (200 kg N ha^-1^) increased N_2_O emissions by up to 27% compared with the sum of N_2_O emissions from individual applications. Davidson et al. [41] reported that cotton residue has a lower C:N ratio than sugarcane, maize, or sorghum; but when cotton residue combined with inorganic N was applied to soil, N_2_O emissions were lower than other crop residues amended treatments. The reasons for the different interactions between N sources are unknown. Our results confirmed previous findings that the residue C:N ratio alone is not always a robust predictor of N_2_O emission, and suggests that other chemical compounds, or even the degradability of the residue C and its different constituents, may need to be considered for predicting N_2_O production when crop residues are added to soils.

Both the maize and wheat crop residue amendements increased cumulative N_2_O emissions in the HAV soil for the treatments of 250 kg N ha^-1^ (MN250 and WN250), and decreased N_2_O cumulative emissions in the treatments with 200 kg N ha^-1^ (MN200 and WN200). The reason for this is most probably that higher N fertilizer application provided more N. The higher nitrate N content of the N250 treatments compared to the N200 treatments also supports this. In our study, available N may be not a limiting factor on N_2_O emissions from soils with 250 kg N ha^-1^, but under the quantity of 200 kg N ha^-1^, available N could become a limiting factor.

N_2_O is generated during microbial nitrification and denitrification, and the responsible microorganisms operate under various optimum conditions. In general, nitrification is dominant at WFPS <60% [41], denitrification becomes dominant at WFPS >60% when NH_4_^+^–N and NO_3_^-^–N are available in the soil [33]. It is possible that denitrification was predominant in our experiment, because the soil WFPS was maintained at 80%. High WFPS (>60%) is favorable for N_2_O production as it decreases oxygen supply and thereby promotes denitrification [42]. In our study, crop residue amendments could stimulate microbial growth and activity, thus promoting oxygen consumption that then created temporary anaerobic microsites, where denitrification is possible. Furthermore, the application of N fertilizer favors nitrification to produce more NO_3_^-^–N, and then more NO_3_^—^N, which also promotes denitrification because denitrifiers prefer NO_3_^-^–N, NO_2_^—^N, and NO to N_2_O for anaerobic respiration. Denitrifies will respire more NO_3_^-^–N than N_2_O, causing more soil N_2_O emissions when soil-available N is relatively abundant [36, 43]. This study showed there were significant positive correlations between soil NH_4_^+^–N (n = 84, r = 0.678, *p*<0.01), NO_3_^-^–N (n = 84, r = 0.384, *p*<0.01) and N_2_O flux in the HAV soil. However, in the OAC soil, only soil NH_4_^+^–N had a significant positive correlation with N_2_O emission flux (n = 84, r = 0.520, *p*<0.01). We do not know the contribution of nitrification and denitrification to the overall N_2_O emissions because we did not make any measurements under acetylene inhibition. Further investigation will be required to identify N_2_O production *via* nitrification in soils under high soil moisture content when crop residues are incorporated.

### Denitrifier gene abundances

Literature reviews have suggested that the abundance of soil denitrifier communities may be factors that affect denitrification [44, 45], with studies reporting that denitrifier communities differ in response to environmental conditions [46, 47]. In our study, there was no significant difference in the total bacterial community abundance between all the treatments, but each slightly increased during the incubation period. The reason for this finding is unclear, but it suggests that the C from crop residues and the N from added N fertilizer are not sufficient to cause a measurable increase in the total bacterial community. Similarly, He et al. [48] found no effect on the total bacterial abundance between treatments receiving both mineral fertilizer and organic manure in comparison to soil receiving no amendments for 16 years in a wheat—maize rotation system.

Addition of crop residues, alone or combined with N fertilizer, resulted in an increase in the *nosZ*-bearing microbial community abundance. This result is in accordance with that of Fabrizzi et al. [4], who found that *nosZ* gene abundances increased significantly in response to all organic carbon treatments over time in anoxic soil microcosms. However, Miller et al. [49] reported that plant residue amendments did not induce a measurable change in the abundances of the *nosZ* gene-bearing community in a laboratory study. These contrasting results may be due to the different concentrations of plant residues used in the respective experiments. Fabrizzi et al. [4] concluded that a high concentration of plant residues is required to increase *nosZ* gene abundance.

In a field study, Kong et al. [40] observed that the number of *nosZ* gene copies were greater in the conventional (annual synthetic fertilizer applications) and low-input systems (synthetic N fertilizer applied in alternate years with cover crop-N incorporated in the years without synthetic N fertilization) than in the organic systems (annual additions of composted manure with a cover crop). However, Hallin et al. [20] found an increase of almost one order of magnitude in the number of *nosZ* gene copies in systems receiving organic fertilizer (solid cattle manure and sewage sludge) compared to that receiving only ammonium sulfate fertilizer. These inconsistent results suggest that *nosZ* gene copies from denitrifiers may be affected by different C sources and environmental factors. Previous studies indicate that some soil microorganism communities adapt to using one type of C source, simple or complex, and will preferentially use that C source. The different soils studied under variable conditions in the different experiments may contain different denitrifiers that are adapted to using simple or complex C sources due to the diverse species targeted by the *nosZ* PCR primers [50]. In our study, variance analysis shows that soil types significantly affect nosZ gene abundance (*P*<0.001).

In our study, the *nirS* gene copy numbers in the treatments with crop residues were significantly higher than in the treatments without crop residues over the course of incubation for both soils. This result was in accordance with previous studies. Tatti et al. [34] reported that crop residue amendments could result in significant increases in *nirS* gene abundance, although Fabrizzi et al. [4] reported that the addition of plant residues had no significant impact on the abundance of *nirS*-bearing denitrifiers. In a field study, Dandie et al. [19] found that there was no difference in the number of *nirS* gene copies between soils in which the crop residues were returned to the field and the crop residues were removed. These inconsistent results could be explained by differences in environmental conditions and N sources used in the respective experiments, and may also be due to the use of different PCR primer sets. We found that a single N fertilizer application did not result in a significant increase in *nirS* gene abundance compared to the CK. N fertilizer application did not obviously impact *nirS* abundance, possibly because the denitrifiers bearing the *nirS* gene were not sensitive to N fertilizer changes in our study. There were no correlations between *nosZ*, *nirS* gene abundances and N_2_O flux in both soils.

## Conclusions

In our study, addition of crop residue resulted in different influence on N_2_O emission in both soils under high soil moisture content (80%WFPS). In the HAV soil, when the quantity of N fertilizer was 250 kg N ha^-1^, the addition of maize or wheat residue increased the cumulative N_2_O emissions compared to the N fertilizer-only treatments, but maize or wheat residue decreased the cumulative N_2_O emissions under fertilizer of 200 kg N ha^-1^ condition. In the OAC soil, wheat residue application increased the cumulative N_2_O emission, but maize residue decreased the cumulative N_2_O emission compared to N fertilizer-only treatments regardless of the quantity of used N fertilizer. Addition of crop residues, either alone or combined with urea, resulted in an increase in the abundances of the *nosZ* and *nirS* genes. But the relationship between N_2_O emission and the denitrifier gene abundances remains unclear, indicating that changes in the denitrifier gene abundances are not the main factors influencing N_2_O emission in our study.

The results of our research implied the current practice of crop residues returning to agricultural soil and fertilization under high soil moisture content would increased N_2_O emission in North China Plain. It should be stressed that the experiments conducted in this study relied on short-term laboratory incubations, and that long-term field experiments are necessary for verification of the results.

## References

[1] IPCC. Climate Change 2007-Mitigation of climate change: Working Group III Contribution to the fourth assessment report of the IPCC: Cambridge University Press; 2007.

[2] Kumar K, Goh KM. Crop residues and management practices: effects on soil quality, soil nitrogen dynamics, crop yield, and nitrogen recovery. Adv Agron. 1999;68:197–319.

[3] Rizhiya EY, Boitsova L, Buchkina N, Panova G. The influence of crop residues with different C: N ratios on the N2O emission from a loamy sand soddy-podzolic soil. Eurasian Soil Sci. 2011;44(10):1144–51.

[4] Fabrizzi K, Garcıa F, Costa J, Picone L. Soil water dynamics, physical properties and corn and wheat responses to minimum and no-tillage systems in the southern Pampas of Argentina. Soil Till Res. 2005;81(1):57–69.

[5] Shan J, Yan X. Effects of crop residue returning on nitrous oxide emissions in agricultural soils. Atmos Environ. 2013;71:170–5.

[6] Yao Z, Zheng X, Wang R, Xie B, Butterbach-Bahl K, Zhu J. Nitrous oxide and methane fluxes from a rice—wheat crop rotation under wheat residue incorporation and no-tillage practices. Atmos Environ. 2013;79:641–9.

[7] Zou J, Huang Y, Lu Y, Zheng X, Wang Y. Direct emission factor for N_2_O from rice—winter wheat rotation systems in southeast China. Atmos Environ. 2005;39(26):4755–65.

[8] Jiang D, Zhuang D, Fu J, Huang Y, Wen K. Bioenergy potential from crop residues in China: Availability and distribution. Renew Sust Energ Rev. 2012;16(3):1377–82.

[9] Ma J, Ma E, Xu H, Yagi K, Cai Z. Wheat straw management affects CH_4_ and N_2_O emissions from rice fields. Soil Biol Biochem. 2009;41(5):1022–8.

[10] Ma W, Bedard-Haughn A, Siciliano S, Farrell R. Relationship between nitrifier and denitrifier community composition and abundance in predicting nitrous oxide emissions from ephemeral wetland soils. Soil Biol Biochem. 2008;40(5):1114–23.

[11] Liu C, Wang K, Meng S, Zheng X, Zhou Z, Han S, et al Effects of irrigation, fertilization and crop straw management on nitrous oxide and nitric oxide emissions from a wheat—maize rotation field in northern China. Agr Ecosyst Environ. 2011;140(1):226–33.

[12] Zhen L, Zoebisch MA, Chen G, Feng Z. Sustainability of farmers' soil fertility management practices: A case study in the North China Plain. J Environ Manage. 2006;79(4):409–19. 16337082 10.1016/j.jenvman.2005.08.009

[13] Ju X-T, Xing G-X, Chen X-P, Zhang S-L, Zhang L-J, Liu X-J, et al Reducing environmental risk by improving N management in intensive Chinese agricultural systems. P Natl Acad Sci USA. 2009;106(9):3041–6. 10.1073/pnas.0813417106 PMC2644255 19223587

[14] He P, Li S, Jin J, Wang H, Li C, Wang Y, et al Performance of an optimized nutrient management system for double-cropped wheat-maize rotations in North-Central China. Agron J. 2009;101(6):1489–96.

[15] Wang Y, Wang E, Wang D, Huang S, Ma Y, Smith CJ, et al Crop productivity and nutrient use efficiency as affected by long-term fertilisation in North China Plain. Nutr Cycl Agroecosys. 2010;86(1):105–19.

[16] Zhao R-F, Chen X-P, Zhang F-S, Zhang H, Schroder J, Römheld V. Fertilization and nitrogen balance in a wheat—maize rotation system in North China. Agron J. 2006;98(4):938–45.

[17] Chen Z, Hou H, Zheng Y, Qin H, Zhu Y, Wu J, et al Influence of fertilisation regimes on a nosZ-containing denitrifying community in a rice paddy soil. J Sci Food Agr. 2012;92(5):1064–72. 21796637 10.1002/jsfa.4533

[18] Shelp ML, Beauchamp EG, Thurtell GW. Nitrous oxide emissions from soil amended with glucose, alfalfa, or corn residues. Commun Soil Sci Plan. 2000;31(7–8):877–92.

[19] Dandie CE, Burton DL, Zebarth BJ, Henderson SL, Trevors JT, Goyer C. Changes in bacterial denitrifier community abundance over time in an agricultural field and their relationship with denitrification activity. Appl Environ Microb. 2008;74(19):5997–6005. 10.1128/AEM.00441-08 PMC2565952 18689522

[20] Hallin S, Jones CM, Schloter M, Philippot L. Relationship between N-cycling communities and ecosystem functioning in a 50-year-old fertilization experiment. ISME J. 2009;3(5):597–605. 10.1038/ismej.2008.128 19148144

[21] Miller MN, Dandie CE, Zebarth BJ, Burton DL, Goyer C, Trevors JT. Influence of carbon amendments on soil denitrifier abundance in soil microcosms. Geoderma. 2012;170:48–55.

[22] Li XY, Gong JD. Effects of different ridge: furrow ratios and supplemental irrigation on crop production in ridge and furrow rainfall harvesting system with mulches. Agr Water Manage. 2002;54(3):243–54.

[23] Sharma P, Abrol V, Sharma R. Impact of tillage and mulch management on economics, energy requirement and crop performance in maize—wheat rotation in rainfed subhumid inceptisols, India. Eur J Agron. 2011;34(1):46–51.

[24] Smith MS, Tiedje JM. Phases of denitrification following oxygen depletion in soil. Soil Biol Biochem. 1979;11(3):261–7.

[25] Van Soest PJ. Use of detergents in the analysis of fibrous feeds. 2. A rapid method for the determination of fiber and lignin. J Asso Off Agr Chem. 1963; 46:546–51.

[26] Suzuki MT, Taylor LT, DeLong EF. Quantitative analysis of small-subunit rRNA genes in mixed microbial populations via 5′-nuclease assays. Appl Environ Microb. 2000;66(11):4605–14. 10.1128/aem.66.11.4605-4614.2000 PMC92356 11055900

[27] Henry S, Bru D, Stres B, Hallet S, Philippot L. Quantitative detection of the nosZ gene, encoding nitrous oxide reductase, and comparison of the abundances of 16S rRNA, narG, nirK, and nosZ genes in soils. Appl Environ Microb. 2006;72(8):5181–9. 10.1128/AEM.00231-06 PMC1538733 16885263

[28] Frimpong K, Baggs E. Do combined applications of crop residues and inorganic fertilizer lower emission of N2O from soil? Soil Use Manage. 2010;26(4):412–24.

[29] Huang Y, Zou J, Zheng X, Wang Y, Xu X. Nitrous oxide emissions as influenced by amendment of plant residues with different C: N ratios. Soil Biol Biochem. 2004;36(6):973–81.

[30] Baggs EM, Rees RM, Smith KA, Vinten AJA. Nitrous oxide emission from soils after incorporating crop residues. Soil Use Manage. 2000;16(2):82–7. 10.1111/j.1475-2743.2000.tb00179.x

[31] Kaiser E-A, Kohrs K, Kücke M, Schnug E, Heinemeyer O, Munch J. Nitrous oxide release from arable soil: importance of N-fertilization, crops and temporal variation. Soil Biol Biochem. 1998;30(12):1553–63.

[32] Eichner MJ. Nitrous oxide emissions from fertilized soils: summary of available data. J Environ Qual. 1990;19(2):272–80.

[33] Lemke R, Izaurralde R, Malhi S, Arshad M, Nyborg M. Nitrous oxide emissions from agricultural soils of the Boreal and Parkland regions of Alberta. Soil Sci Soc Am J. 1998;62(4):1096–102.

[34] Tatti E, Goyer C, Chantigny M, Wertz S, Zebarth BJ, Burton DL, et al Influences of over winter conditions on denitrification and nitrous oxide-producing microorganism abundance and structure in an agricultural soil amended with different nitrogen sources. Agr Ecosyst Environ. 2014;183:47–59.

[35] Azam F, Müller C, Weiske A, Benckiser G, Ottow J. Nitrification and denitrification as sources of atmospheric nitrous oxide—role of oxidizable carbon and applied nitrogen. Biol Fert Soils. 2002;35(1):54–61. 10.1007/s00374-001-0441-5

[36] Li X, Hu F, Shi W. Plant material addition affects soil nitrous oxide production differently between aerobic and oxygen-limited conditions. Appl Soil Ecol. 2013;64:91–8.

[37] Vinten AJ, Whitmore A, Bloem J, Howard R, Wright F. Factors affecting N immobilisation/mineralisation kinetics for cellulose-, glucose-and straw-amended sandy soils. Biol Fert Soils. 2002;36(3):190–9.

[38] Rousk J, Bååth E. Fungal and bacterial growth in soil with plant materials of different C/N ratios. FEMS Microbiol Ecol. 2007;62(3):258–67. 10.1111/j.1574-6941.2007.00398.x 17991019

[39] Begum N, Guppy C, Herridge D, Schwenke G. Influence of source and quality of plant residues on emissions of N2O and CO2 from a fertile, acidic Black Vertisol. Biol Fert Soils. 2013;50(3):499–506. 10.1007/s00374-013-0865-8

[40] Kong AYY, Hristova K, Scow KM, Six J. Impacts of different N management regimes on nitrifier and denitrifier communities and N cycling in soil microenvironments. Soil Biol Biochem. 2010;42(9):1523–33. 10.1016/j.soilbio.2010.05.021 21339865 PMC3040239

[41] Davidson EA, Keller M, Erickson HE, Verchot LV, Veldkamp E. Testing a Conceptual Model of Soil Emissions of Nitrous and Nitric Oxides Using two functions based on soil nitrogen availability and soil water content, the hole-in-the-pipe model characterizes a large fraction of the observed variation of nitric oxide and nitrous oxide emissions from soils. BioScience. 2000;50(8):667–80.

[42] Ruser R, Flessa H, Russow R, Schmidt G, Buegger F, Munch J. Emission of N 2 O, N 2 and CO 2 from soil fertilized with nitrate: effect of compaction, soil moisture and rewetting. Soil Biol Biochem. 2006;38(2):263–74.

[43] Robertson G, Tiedje J. Nitrous oxide sources in aerobic soils: nitrification, denitrification and other biological processes. Soil Biol Biochem. 1987;19:187–93.

[44] Philippot L, Hallin S. Finding the missing link between diversity and activity using denitrifying bacteria as a model functional community. Curr Opin Microbiol. 2005;8(3):234–9. 15939345 10.1016/j.mib.2005.04.003

[45] Wallenstein MD, Myrold DD, Firestone M, Voytek M. Environmental controls on denitrifying communities and denitrification rates: insights from molecular methods. Eco Appl. 2006;16(6):2143–52. 10.1890/1051-0761(2006)016[2143:ecodca]2.0.co;2 17205893

[46] Holtan-Hartwig L, Dörsch P, Bakken LR. Comparison of denitrifying communities in organic soils: kinetics of NO3− and N_2_O reduction. Soil Biol Biochem. 2000;32(6):833–43. 10.1016/S0038-0717(99)00213-8

[47] Cavigelli MA, Robertson GP. The functional significance of denitrifier community composition in a terrestrial ecosystem. Ecology. 2000;81:1402–14.

[48] He F, Chen Q, Jiang R, Chen X, Zhang F. Yield and nitrogen balance of greenhouse tomato (Lycopersicum esculentum Mill.) with conventional and site-specific nitrogen management in Northern China. Nutr Cycl Agroecosys. 2007;77(1):1–14.

[49] Miller MN, Zebarth BJ, Dandie CE, Burton DL, Goyer C, Trevors JT. Crop residue influence on denitrification, N_2_O emissions and denitrifier community abundance in soil. Soil Biol Biochem. 2008;40(10):2553–62. 10.1016/j.soilbio.2008.06.024

[50] Yu Y, Zhang J, Chen W, Zhong W, Zhu T, Cai Z. Effect of land use on the denitrification, abundance of denitrifiers, and total nitrogen gas production in the subtropical region of China. Biol Fert Soils. 2014;50(1):105–13.

